# Habits and beliefs related to food supplements: Results of a survey among Italian students of different education fields and levels

**DOI:** 10.1371/journal.pone.0191424

**Published:** 2018-01-19

**Authors:** Felice Sirico, Salvatore Miressi, Clotilde Castaldo, Rocco Spera, Stefania Montagnani, Franca Di Meglio, Daria Nurzynska

**Affiliations:** Department of Public Health, University of Naples Federico II, Naples, Italy; The Chinese University of Hong Kong, HONG KONG

## Abstract

The increasing availability of food supplements, aggressive media advertising, and common beliefs that these substances have only positive effects on health and sport performance indicate a need for continuous monitoring of this phenomenon. The aim of this study was to investigate the habits and beliefs related to diet supplementation among medical, health professional, and other university/high school students by means of a cross-sectional anonymous survey online. Among the respondents aware of supplements, 37.4% were taking or had taken them in the past. Food supplement use was more common among university students (in particular, those in health professional graduate courses) than high school students. Individual sport practice, rather than team sport, was associated with higher likelihood of food supplement use. Multivitamins were most commonly used, while weight-loss formulations were the least popular. Strikingly, filling nutrient gaps was statistically not considered the main reason for taking food supplements. Instead, they were used to enhance mental performance or enhance well-being. There was statistical evidence that students not enrolled in health or medical professional studies strongly agreed more often than medical students that taking food supplements prevents illness. These results indicate a striking difference between the evidence-based and personal reasons for food supplement use. Arguably, it calls for an improvement in education about diet supplementation and a change in attitude of health care providers to its implementation.

## Introduction

The European Food Safety Authority (EFSA) defines food supplements as concentrated sources of nutrients or other substances with a nutritional or physiological effect, whose purpose is to supplement a normal diet [1]. These substances are produced as pills, capsules, or liquids, and are similar to formulations typical of medicines. They contain concentrated vitamins, minerals, or other substances in specific dosages. The European Union (EU) Directive 2002/46/EC set out labelling requirements and required that the tolerable upper intake levels were set for each vitamin and mineral added as a supplement [2]. This latter task has been delegated to the EFSA and is currently ongoing. Recent report from EFSA [3] summarizes the currently established daily reference values for vitamins and minerals for European population. In S1 Table, we compared these values with the RDI established by the FDA [4].

Vitamins, minerals, and botanical products are among the most commonly used dietary supplements in the United States [5], with energy drinks being alarmingly popular among the adolescents and young adults in recent years [6]. In the EU, products containing vitamins and minerals represent 50% of total sales of food supplements, those containing other substances have 43% market share, while tonics are the least popular (7%) [7]. The sales of the products other than mineral and vitamins, among the 17 member states included in the recent analysis, are highest in Italy. In this country, the probiotics (including Inulin, Lactobacillus acidophilus, Bifidobacterium species, and Yeast species) are the most commercially important products (44% of the market size of other substances), followed by combined formulas (25%). The consumption of the latter is particularly alarming, since the monitoring of the actual dosage of substances in the consumed products may be difficult for unaware users.

In physiological conditions, the supply of the required amount of nutrients is usually met by a normal diet (i.e. a regular diet that includes all foods and meets the energy and nutrient needs of healthy people). Based on the definition of food supplements and considering their physiological effects on different metabolic processes, the decision regarding their use should be taken carefully and be justified by the increased physiological demands or insufficient intake of nutrients from dietary sources. While multivitamins are safe at doses corresponding to RDI in the short and the long term, the adverse effects may occur if single vitamins at high doses are consumed [8]. Indeed, according to published studies, the amount of specific nutrients can exceed dietary reference intakes by as much as 150%–3100% due to unjustified supplementation [9]. About 8%–9% of consumers develop adverse effects like diarrhoea, constipation, gastric pain, headache, nausea, and vomiting. However, the eventuality of more severe adverse effects, such as neurological disturbances, liver toxicity, and interference with other drugs, should be considered [10]. According to a recent retrospective analysis [11], serious medical outcomes accounted for 4.5% of food supplement exposures reported to US Poison Control Center between 2000 and 2012. Overall, exposure to yohimbe and energy products was associated with the greatest toxicity Importantly, the majority of dietary supplement exposures occurred among children younger than 6 years old and were unintentional. Therefore, unregulated consumption of food supplements could represent a risk, rather than a benefit, for health in some people.

The common motivations for the consumption of food supplements are to prevent disease, to enhance mental and general health, to enhance sport performance, and to compensate for dietary deficiencies [12, 13]. The increasing availability of food supplements, aggressive advertising in the media, and the common beliefs that these substances have only positive effects on health, sustained by the Council for Responsible Nutrition [14], indicate a need for continuous monitoring of this phenomenon by medical community. Available data suggest that physician knowledge about food supplements is limited [15]. In a recent study, many interviewed physicians admitted they lack sufficient knowledge regarding food supplement safety and efficacy; moreover, approximately 73% of them did not know how or where to report the adverse effects associated with food supplements in their patients [16]. When coupled with personal assumption that food supplements are mostly safe and effective, the potential for out-of-control diet supplementation and adverse effect development may reach medically relevant scale. In this regard, a scientific approach to the interpretation of available information and adequate education about diet supplementation should be implemented in medical and health professional studies graduate programs to prepare medical doctors and health providers for appropriate patient counselling.

The aim of this study was to investigate habits and beliefs related to food supplements among high school and university students. Data about physical activity, use of food supplements, attitudes associated with food supplement use, and opinions and beliefs about efficacy and safety of diet supplementation were recorded. Overall sample analysis and participant clustering based on type of education or use/nonuse of food supplements were applied. The findings of this study shed light on the factors influencing students' supplement-taking behaviour and highlight the need for a better education and regulations regarding food supplement use.

## Materials and methods

A cross-sectional online survey was conducted among students who speak Italian as their mother tongue. The questionnaire was edited in clear native language and organized into 18 items. The survey (available in original language and translated in English in S1 Survey) was designed and conducted using Google Forms. All students of the University of Naples "Federico II" received a message inviting them to participate, with the link to the survey form, via an institutional e-mail. High school students were recruited by giving the same information to the participants of the School-University Alternation programme from the "Umberto I" High School in Naples. Participation was voluntary and all questionnaires were anonymous. The survey was self-administered via web by each participant.

Questions were designed to assess the following domains: anagraphic data (age, sex), education, physical activity (type, frequency, intensity), use of food supplements, attitudes associated with food supplement use, and beliefs about safety and efficacy of food supplements. Participants were asked to answer multiple choice questions related to awareness about food supplement products and attitudes associated with supplement use (frequency and circumstances of use, source of motivation and information, and observed adverse effects). Questions with a 10-point ordinal scale were asked to investigate how participants rated the overall safety and efficacy of supplementation, and a Likert scale was used to assess opinions about common statements related to food supplementation.

Continuous variables were summarized reporting mean and standard deviation. The Shapiro–Wilk test was used to assess for normality. The Mann–Whitney nonparametric test was adopted to compare means between two independent groups when the assumptions for the t-test were not met. Median values and interquartile ranges were used for ordinal data. Frequencies and percentages of categorical variables were reported and compared using chi-squared statistics. A binomial logistic regression model was used to investigate the relationship between a dichotomous dependent variable and a dichotomous independent variable. All data were analysed using STATA software v.12 (StataCorp. 2011, Stata Statistical Software: Release 12. College Station, TX: StataCorp LP).

## Results

### Demographic data

The questionnaire was completed by 770 participants, of which 195 (25.3%) were high school and 575 (74.7%) were university students. The sample included 405 (52.6%) females and 365 (47.4%) males, and the mean age of the participants was 21.53 years (*SD* = 4.2, range: 13–41).

Only 4% of all the respondents were not knowledgeable about food supplements. Of those participants who were aware of food supplementation products, 37.4% admitted that they were taking them or had taken them. These data were not related to sex, as the "users" group was composed of 154 females and 134 males (χ^2^ = 0.1; *p* = 0.7). Interestingly, correlation with the level of education was evident, with the consumption of food supplements being more common among university students than high school students (241/575 university students vs. 47/195 high school students, χ^2^ = 19.7; *p* < 0.001). When participants were grouped based on the type of studies (medical university studies, health professional university studies, other university/high school studies), food supplement use was acknowledged by 40.7%, 44.8%, and 32.6% of the respondents, respectively.

Students who acknowledged using food supplements were significantly older than those who did not in both sexes (mean age of female users: 23 years, *SD* = 4.75; female nonusers: 20.5 years, *SD* = 3.8, *p* < 0.001; male users: 22.5 years, *SD* = 4.2; male nonusers: 21.1 years, *SD* = 4.0, *p* < 0.001).

### Food supplement consumer habits

**Fig 1:**
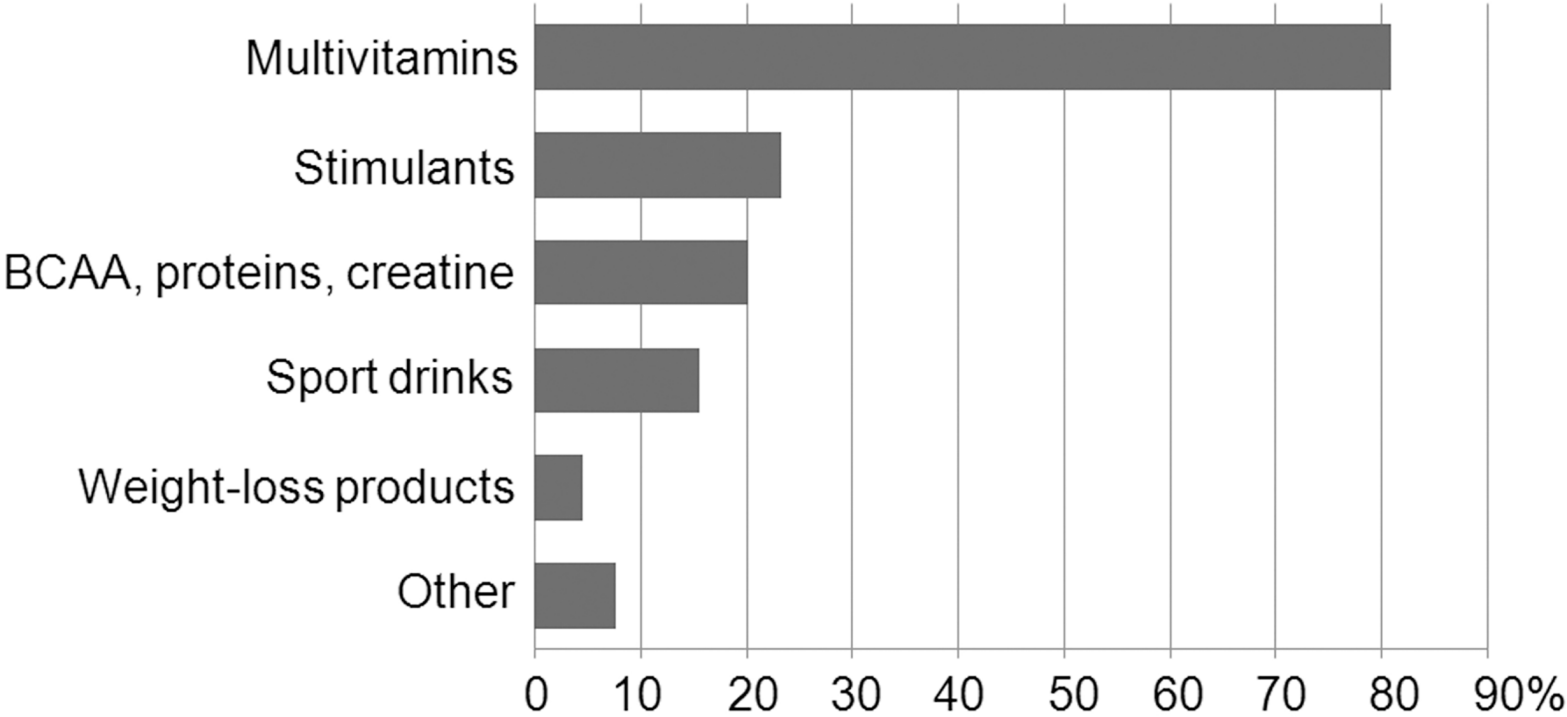
Types of food supplements used by the respondents.

Of those who acknowledged using food supplements, 1.7% consumed them two times a day, while 41.8% took them once daily. The remaining students reported the frequency of use ranging from one to six times per week. Food supplements covered by the survey were divided in six categories: (1) branched-chain amino acids, protein powders, and creatine products, (2) multivitamins, (3) sport drinks, (4) weight-loss products, (5) stimulants (including energy drinks, caffeine, guaranà, and ginseng), and (6) other supplements. Among the categories of food supplements, multivitamins were most commonly used, while weight-loss formulations were the least popular (Fig 1). Moreover, 68% of the students used more than one type of food supplement.

**Fig 2:**
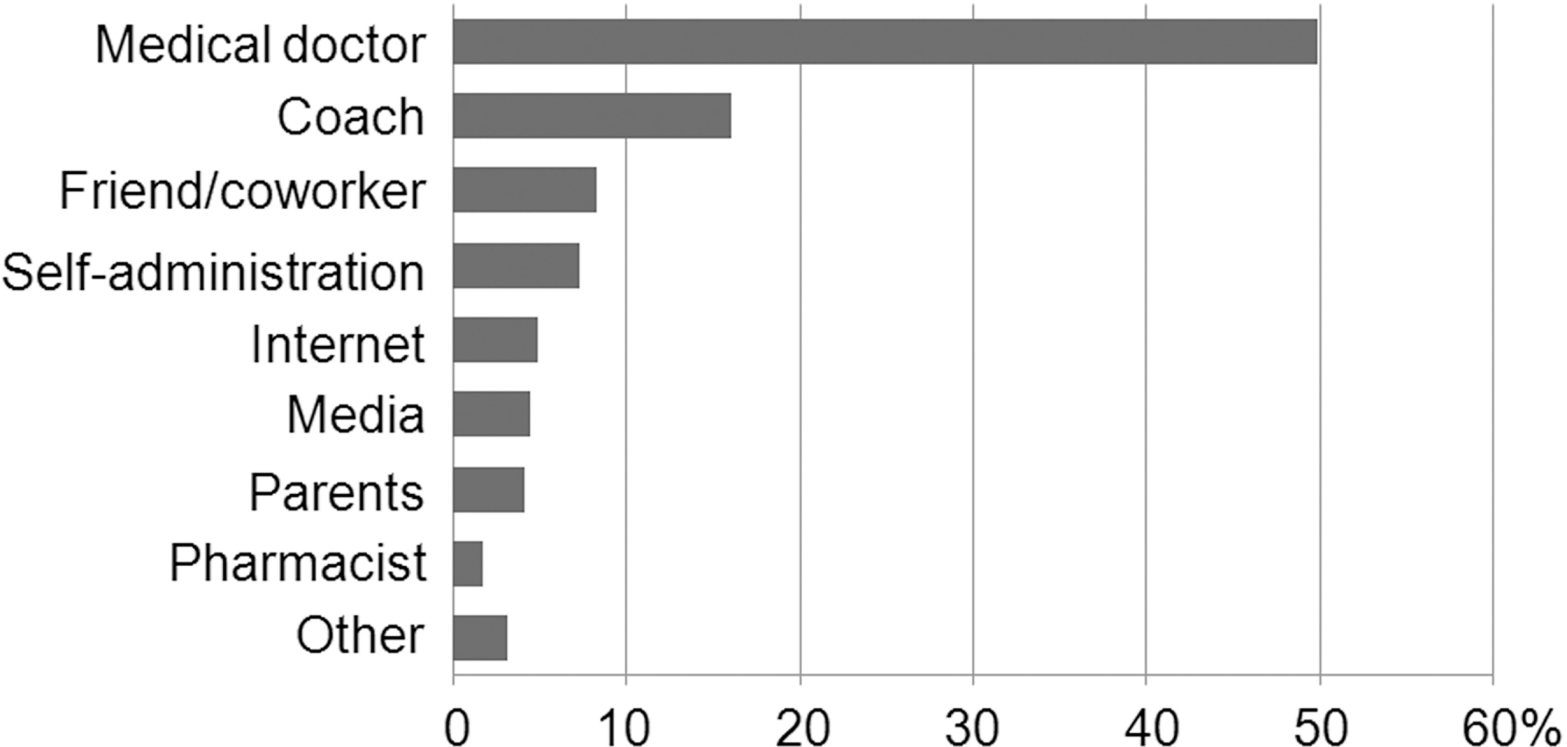
Source of influence on the respondents' decision to use food supplements.

Nearly half of the respondents who used food supplements admitted that they followed a medical doctor's recommendation; the second most common source of influence on the decision to use food supplements was a sport coach's advice (Fig 2). Importantly, similar numbers of students admitted taking advice from friends/co-workers or the media/internet, or making the decision by themselves.

**Fig 3:**
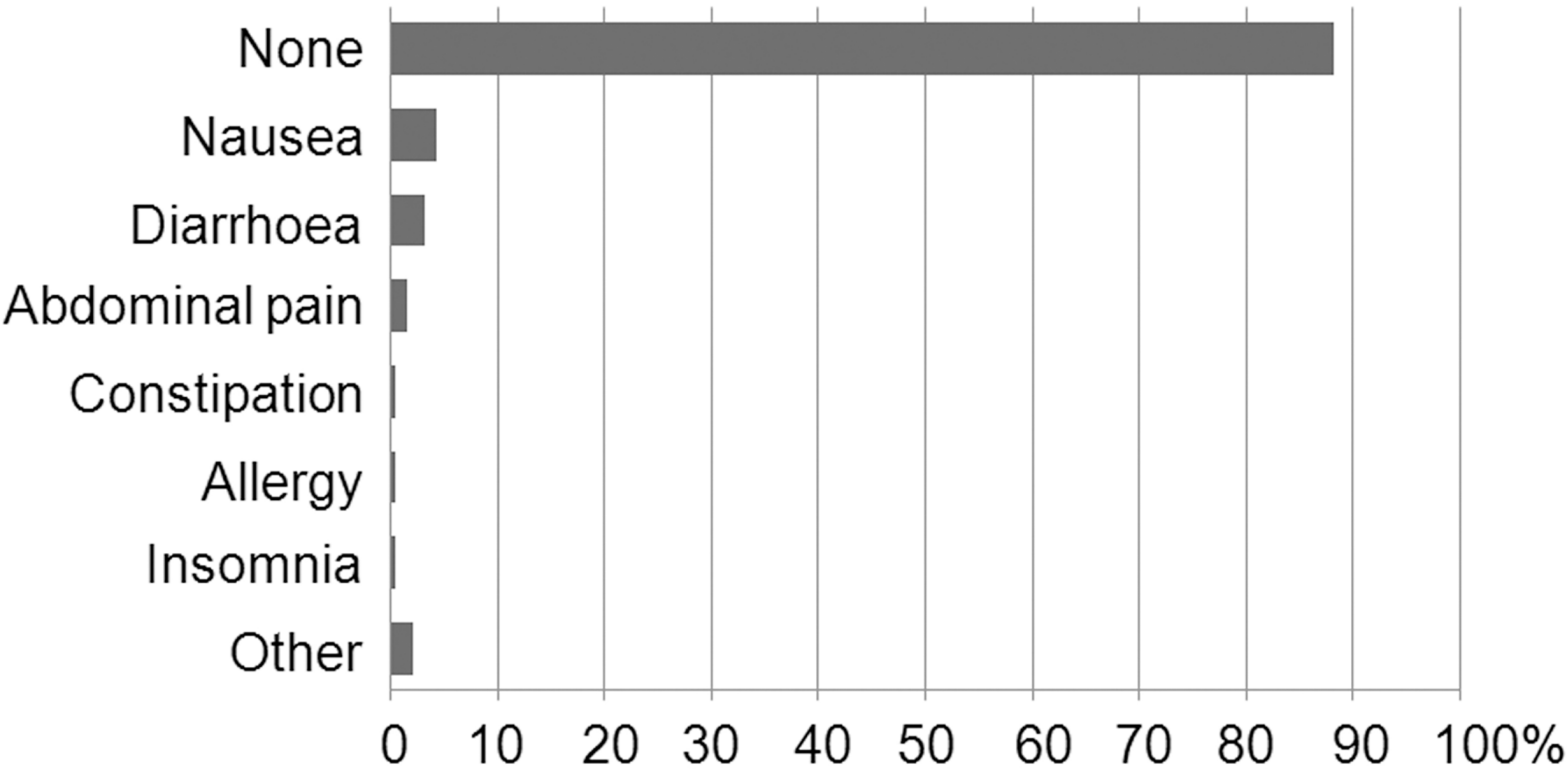
Adverse effects observed by the users of food supplements.

Of the students who used food supplements, 88.2% did not report any of the recognized adverse effects of supplement use (Fig 3). Those who did, complained mainly of gastrointestinal problems (diarrhoea, constipation, nausea, abdominal pain). One respondent reported insomnia and another suffered a hypersensitivity reaction.

Among all the respondents, 53.5% (412 students) engaged in regular sport activity, with a mean of 1.7 training sessions of 45 minutes a week. The use of food supplements, however, was not exclusive to those who practiced sport. Indeed, 158 students of 412 who engaged regularly in sport activity acknowledged consuming supplementation products (38.4%) and a similar rate was evident for those who took these products even though they did not practise sport (130/358 participating students; 36.3%). To investigate further the relation between the food supplement use and sport, those respondents who declared that they were physically active were grouped based on the type of sport they practised. Owing to heterogeneity of the answers, students were divided into those who engaged in individual sport or participated in team sport. The rate of food supplement use was significantly higher in the individual than in the team sport group (χ^2^ = 4.6, *p* = 0.03). Next, logistic regression was performed to explain the possible role of independent variables on the dependent dichotomous variable and measure association between the sport type and use of food supplements. We found that individual sport practice was associated with higher likelihood of food supplement use, with an odds ratio of 1.8.

### General beliefs about food supplements

The most common reasons for the actual or possible use of food supplements chosen by the participants were to enhance sport performance and enhance well-being (Table 1). However, food supplement users and nonusers differed in this regard (Table 2). In particular, the main reason indicated by the nonusers was that diet supplementation could improve sport performance and be used as a substitute for a skipped meal, while those who actually used food supplements frequently declared that the main reason was to enhance mental performance or enhance well-being. Importantly, in both groups, filling nutrient gaps was statistically not considered the main reason for taking food supplements.

**Table 1:** Main reason for the use of food supplements selected by the respondents.

Reason	N	%
Enhance sport performance	321	41.7
Enhance well-being	220	28.6
Enhance mental/cognitive performance	67	8.7
Gain weight	29	3.8
Lose weight	29	3.8
Supplement dietary deficiencies	22	2.9
Substitute a meal	17	2.2
No response	65	8.4

**Table 2:** Main reason for the use of food supplements selected by the users and nonusers.

		Users	Nonusers	Total
Enhance sport performance	N	57	264	321
	Row %	17.8	82.2	100
	Column %	19.8	54.8	41.7
Enhance well-being	N	133	87	220
	Row %	60.4	39.6	100
	Column %	46.2	18.0	28.6
Enhance mental/cognitive performance	N	56	11	67
	Row %	83.6	16.4	100
	Column %	19.4	2.3	8.7
Gain weight	N	9	20	29
	Row %	31.0	69.0	100
	Column %	3.1	4.1	3.8
Lose weight		14	15	29
	Row %	48.3	51.7	100
	Column %	4.9	3.1	3.8
Supplement dietary deficiencies	N	10	12	22
	Row %	45.4	54.6	100
	Column %	3.5	2.5	2.9
Substitute a meal	N	2	15	17
	Row %	11.8	88.2	100
	Column %	0.7	3.1	2.2
No response	N	7	58	65
	Row %	10.8	89.2	100
	Column %	2.4	12.0	8.4
Total	N	288	482	770
	Row %	37.4	62.6	100
	Column %	100	100	100
χ^2^ = 190.9; *p* = 0.0				

Opinions regarding the positive impact of food supplementation on physical performance in sport activities and the negative impact on health in general was evaluated using a 10-point ordinal scale (1 = no impact on sport performance or completely safe to health, 10 = the highest impact on sport performance or extremely harmful to health). In the whole sample, the median value in both evaluations was 5. When the type of studies was considered, the median values for impact on sport performance was 5 (interquartile range [IQR] 3–7) for medical, 5 (IQR 3–6) for health professional, and 6 (IQR 4–7) for other university studies and high school students; the respective median values for health effects were 4 (IQR 2–6), 4.5 (IQR 3–6.5), and 5 (IQR 3–7). The participants were then asked to indicate on an ordinal scale (strongly agree, agree, neither agree nor disagree, disagree, or strongly disagree) their opinion or belief regarding the following statements about diet supplementation: food supplements are necessary at all ages, food supplements are essentially harmless, regular food supplement intake can prevent cancer, regular food supplement intake can prevent chronic diseases. The answers were analysed according to the type of respondents' education (Table 3). There was statistical evidence that high school and other university students (those enrolled in nonmedical and nonhealth professional studies) strongly agreed more often than medical students that taking food supplements can prevent illness.

**Table 3:** Respondents' beliefs regarding food supplements.

		Necessary at all ages				Essentially harmless				Prevent chronic diseases				Prevent cancer			
		MU	HPU	OU/HS	Total	MU	HPU	OU/HS	Total	MU	HPU	OU/HS	Total	MU	HPU	OU/HS	Total
**SA+A**	N	34	10	44	88	97	31	107	235	39	13	61	113	13	7	27	47
	Row %	38.6	11.4	50.0	100	41.3	13.2	45.5	100	34.5	11.5	54.0	100	27.7	14.9	57.4	100
	Column %	11.4	9.5	12.0	11.4	32.7	29.5	29.1	30.5	13.1	12.4	16.6	14.7	4.4	6.7	7.3	6.1
**Neutral**	N	51	21	65	137	83	35	133	251	116	33	174	323	79	25	148	252
	Row %	37.2	15.3	47.4	100	33.1	13.9	53.0	100	35.9	10.2	53.9	100	31.3	9.9	58.7	100
	Column %	17.2	20.0	17.7	17.8	27.9	33.3	36.1	32.6	39.1	31.4	47.3	41.9	26.6	23.8	40.2	32.7
**D+SD**	N	210	72	256	538	115	39	126	280	140	59	131	330	203	73	191	467
	Row %	39.0	13.4	47.6	100	41.1	13.9	45.0	100	42.4	17.9	39.7	100	43.5	15.6	40.9	100
	Column %	70.7	68.6	69.6	69.9	38.7	37.1	34.2	36.4	47.1	56.2	35.6	42.9	68.4	69.5	51.9	60.6
**No response**	N	2	2	3	7	2	0	2	4	2	0	2	4	2	0	2	4
	Row %	28.6	28.6	42.8	100	50.0	0	50.0	100	50.0	0	50.0	100	50.0	0	50.0	100
	Column %	0.7	1.9	0.8	0.9	0.7	0	0.5	0.5	0.7	0	0.5	0.5	0.7	0	0.5	0.5
**Total**	N	297	105	368	770	297	105	368	770	297	105	368	770	297	105	368	770
	Row %	38.6	13.6	47.8	100	38.6	13.6	47.8	100	38.6	13.6	47.8	100	38.6	13.6	47.8	100
	Column %	100	100	100	100	100	100	100	100	100	100	100	100	100	100	100	100
**Statistics**		χ^2^ = 10.5; *p* = 0.4				χ^2^ = 8.9; *p* = 0.5				χ^2^ = 22.8; *p* = 0.0				χ^2^ = 33.0; *p* = 0.0			

D+SD, disagree and strongly disagree; HPU, health professional university students; MU, medical university students; OU/HS = other university/high school students; SA+A, strongly agree and agree.

## Discussion

Data on the usage of food supplements in Europe are still limited, with the main data available from commercial market analysis rather than consumer surveys. Our survey, with its data on beliefs and attitudes related to food supplementation, falls perfectly within the domain of current interests and concerns of the European Food Safety Authority [17], which published an open public consultation, active till June 1, 2017, aimed to collect the views of citizens on nutrition and health claims made on foods and how plant substances used in foods are regulated in the EU. In particular, the survey investigated how potential consumers of products marketed with nutrition or health claims understand these claims and other nutrition information provided on the label, how they perceive the healthiness of foods making such claims, and what specific elements drive their food choices.

To our knowledge, this study is the first to explore the attitudes and beliefs of students of different education fields and levels in how food supplements are perceived and used. The results demonstrate that this group is interested in diet supplementation. Indeed, nearly 40% of the participants were taking at least one food supplement product at the time of survey or had taken them previously. It was our intention, reflected in the purposeful design of the survey, to consider also occasional and seasonal use, as other consumer surveys tend to report artificially low results including only regular and ongoing use of food supplements [18].

The "statistical user" has the following demographic characteristics: white, older, female, education beyond high school, higher income [19]. Contrary to other studies that reported more frequent use of food supplementation by women [18, 19], our survey did not reveal any significant difference between sexes. However, users of food supplements were slightly older than nonusers and were mainly graduate health professional students. Most food supplement users took multivitamins, and many took a variety of products. The consumption of vitamins and minerals, regardless of the intake from food alone, is a common attitude in different age groups, particularly in individuals who have a significant concern for their overall health [20] and in those from families with a high socioeconomic level [21, 22].

Reports indicate that the people most likely to use food supplements are those who are least likely to need them [23]. Accordingly, for a significant number of users, intake of folic acid, vitamin D, calcium, and iron often exceeds the recommended daily allowance [9]. Self-administration of several food supplements can have serious adverse effects, caused by sympathomimetic activity of caffeine or yohimbe products, and interactions between drugs and herbs (e.g. the additive anticoagulant/antiplatelet effects of NSAIDs taken with ginkgo) [24] or alcohol and energy drinks [25]. In this connection, it is important to note that the adverse events related to the consumption of food supplements, even if infrequent among the participants of our survey, are rarely reported to pharmacovigilance authorities [26].

Remarkably, according to our survey, only 50% of food supplement users were advised by a medical doctor, while others were often influenced by sport coaches, friends, or the internet and other media. In a recent study in the general population [22], less than a quarter of supplements used by adults were recommended by a physician or health care provider. In fact, the media, in the form of magazine and television advertisements, are perceived to be a powerful influence on a person's decision to use supplements [27].

Sport practise is generally considered to be a healthy habit. In this regard, some studies indicated that users of food supplements were more likely than nonusers to engage in sport activity, suggesting that supplement use is part of an overall attitude toward healthy living [18, 22]. Such a correlation was not evident in our survey, as a similar rate of food supplement users was observed among those who practised and did not practise sport. However, team sport was a preventive factor for diet supplementation. The psychosocial determinants of such influence of team sport among Italian students should be investigated further and could be exploited in practise to monitor food supplementation use in young adults.

Observations from a pilot ethnographic study of dietary supplementation among Americans identified a wide range of overlapping and interactive motivations regarding supplement use, many being pragmatic, strategic, and ideological [28]. The primary reasons given for supplement use by the participants of our survey were to enhance sport performance and overall health and wellness. Keeping in mind the current definition of food supplements, edited by the EFSA, it was interesting to note that the main reason for food supplement use, as indicated by the participants of our survey, was not to fill nutrient gaps. In particular, the analysis of the answers indicated that those who took food supplements did it for general well-being or to enhance mental performance, even though a specific dietary deficiency that could reduce their health or cognitive capacity and necessitate the use of supplementation products was presumably not identified. The nonusers commonly believed that the main reason for taking food supplements was to enhance sport performance. This agrees with the observation by Nichter and Thompson [28] that food supplements are consumed 'for my wellness, not just my illness'. These results indicate an important difference between the intended scientific and actual personal reasons for food supplement use. Arguably, these results call for improving education about diet supplementation and changing the attitude of health care providers toward its implementation. Considering the powerful influence of media on a person's decision to use supplements [27], these data also indicate the need for stricter regulation of food supplementation advertising and commerce.

Several previous surveys and analyses examining supplement-taking behaviour concluded that users believed more strongly than nonusers that taking food supplements would help them to be healthy [22, 27], either by actually enhancing health or by reducing harm from an unhealthy lifestyle or environment [28]. In our survey, the analysis of beliefs underlying food supplement use revealed differences between students receiving different types of education in relation to the notion that taking food supplements acts as insurance against possible chronic disease and cancer. In particular, high school and other university students (i.e. those not enrolled in health or medical professional studies) believed more strongly than medical students that taking food supplements can prevent illness. Although food supplements have their place in preventing identified nutritional deficiencies, there is no sufficient evidence to support diet supplementation in the primary prevention of cause-specific death, incidence of cardiovascular disease and incidence of cancer [29]. Nonetheless, strong pressure from peers and the media exists to unnecessarily encourage food supplement use. These beliefs and attitudes should be modified by introducing precise regulations regarding food supplement labelling and advertising, together with developing a higher level of competency among doctors and health care providers in nutrition and disease prevention counselling.

## Conclusions

In conclusion, the results of our survey suggest that beliefs and attitudes of surveyed students of medical and health professional programs are rarely based on medical evidence. In future, these professional figures should be able to make recommendations supported by scientific data and food safety authority regulations, rather than by expressing their personal beliefs and transmitting their own attitudes to their patients. It is advisable to include specific courses or lectures covering the indications, interactions, and potential adverse effects of common food supplements in health care studies programs. As scientific knowledge about this topic is bound to increase in the coming years, organization of continuous medical education courses for clinicians and therapists should be encouraged.

## Supporting information

S1 Table Reference values for daily intakes of the vitamins and minerals which are essential in human nutrition. (PDF)

S1 Survey The questionnaire used in the survey, in original and English language. (PDF)
