# Supplementary material for: Habits and beliefs related to food supplements: Results of a survey among Italian students of different education fields and levels
Source: PLoS One. 2018 Jan 19;13(1):e0191424. doi: 10.1371/journal.pone.0191424 (PMC5774790; doi:10.1371/journal.pone.0191424)
Supplement: S1 Table — (PDF) [file pone.0191424.s001.pdf]

Reference values for daily intakes of the vitamins and minerals which are essential in human nutrition.

<table>
<tr><th>Supplement</th><th colspan="3">PRI or AI according to EFSA [3]</th><th>RDI according to FDA [4]</th></tr>
<tr><td>Biotin (µg)</td><td colspan="3">40</td><td>30</td></tr>
<tr><td rowspan="4">Calcium (mg)</td><td rowspan="2">18-24y</td><td>M</td><td>1000</td><td rowspan="4">1300</td></tr>
<tr><td>F</td><td>1000</td></tr>
<tr><td rowspan="2">≥25y</td><td>M</td><td>950</td></tr>
<tr><td>F</td><td>950</td></tr>
<tr><td>Chloride (mg)</td><td colspan="3">800</td><td>2300</td></tr>
<tr><td>Choline (mg)</td><td colspan="3">400</td><td>550</td></tr>
<tr><td>Chromium (µg)</td><td colspan="3">40</td><td>35</td></tr>
<tr><td rowspan="2">Copper (mg)</td><td>M</td><td colspan="2">1.6</td><td rowspan="2">0.9</td></tr>
<tr><td>F</td><td colspan="2">1.3</td></tr>
<tr><td rowspan="2">Fluoride (mg)</td><td>M</td><td colspan="2">3.4</td><td rowspan="2">-</td></tr>
<tr><td>F</td><td colspan="2">2.9</td></tr>
<tr><td>Folate (µg DFE)</td><td colspan="3">330</td><td>400</td></tr>
<tr><td>Iodine (µg)</td><td colspan="3">150</td><td>150</td></tr>
<tr><td rowspan="3">Iron (mg)</td><td>M</td><td colspan="2">11</td><td rowspan="3">18</td></tr>
<tr><td rowspan="2">F</td><td>premenop</td><td>16</td></tr>
<tr><td>postmenop</td><td>11</td></tr>
<tr><td rowspan="2">Magnesium (mg)</td><td>M</td><td colspan="2">350</td><td rowspan="2">420</td></tr>
<tr><td>F</td><td colspan="2">300</td></tr>
<tr><td>Manganese (mg)</td><td colspan="3">3</td><td>2.3</td></tr>
<tr><td>Molybdenum (µg)</td><td colspan="3">65</td><td>45</td></tr>
<tr><td>Niacin (mg NE)</td><td colspan="3">1.6/megajoul</td><td>16</td></tr>
<tr><td>Panthotenic acid (mg)</td><td colspan="3">5</td><td>5</td></tr>
<tr><td>Phosphorus (mg)</td><td colspan="3">550</td><td>1250</td></tr>
<tr><td>Potassium (mg)</td><td colspan="3">3500</td><td>4700</td></tr>
<tr><td>Riboflavin (mg)</td><td colspan="3">1.6</td><td>1.3</td></tr>
<tr><td>Selenium (µg)</td><td colspan="3">70</td><td>55</td></tr>
<tr><td>Thiamin (mg)</td><td colspan="3">0.1/megajoul</td><td>1.2</td></tr>
</table>

Vitamin A (µg)	M	750	900
	F	650	
Vitamin B12 (µg)	4		2.4
Vitamin B6 (mg)	M	1.7	1.7
	F	1.6	
Vitamin C (mg)	M	110	90
	F	95	
Vitamin D (µg)	15		20
Vitamin E (mg)	M	13	15
	F	11	
Vitamin K (µg)	70		120
Zinc (mg)	M	9.4-16.3*	11
	F	7.5-12.7*	

AI, average intake (the average observed daily level of intake by a population group of apparently healthy people that is assumed to be adequate; this value is used when the PRI could not be established); F, female; M, male; DFE, dietary folate equivalents; NE, niacin equivalent; PRI, population reference intake (the level of nutrient intake that is adequate for virtually all people in a population group); RDI, reference daily intake.

* depending on the level of phytate intake.
