# Supplementary material for: Habits and beliefs related to food supplements: Results of a survey among Italian students of different education fields and levels
Source: PLoS One. 2018 Jan 19;13(1):e0191424. doi: 10.1371/journal.pone.0191424 (PMC5774790; doi:10.1371/journal.pone.0191424)
Supplement: S1 Survey — (PDF) [file pone.0191424.s002.pdf]

Abitudini e opinioni degli studenti italiani in merito agli integratori alimentari

1. Età: ....anni

2. Sesso:
☐ Maschio
☐ Femmina

3. Livello di istruzione:
☐ Studente Universitario – Corso di Laurea in Medicina e Chirurgia
☐ Studente Universitario – corso di laurea in professioni sanitarie
☐ Studente Universitario – altro corso di laurea
☐ Studente delle scuole superiori

4. Hai praticato attività sportiva per almeno tre mesi consecutivi negli ultimi 12 mesi?
□ Si, (specificare sport ...) □ No

5. Se sì, quanti allenamenti a settimana?
□1 □2 □3 □4 □ 5 □6 □7 □>7 (specificare …)

6. Se sì, quanto dura in media un allenamento (in minuti)?
□ 30 □45 □60 □90 □120 □ >120 (specificare ...)

7. Sai cosa sono gli integratori alimentari?
□ Si □ No

8. Attualmente assumi oppure hai assunto in passato integratori alimentari?
□ Si □ No

Se hai risposto **SI** alla domanda precedente, rispondi a **tutte** le seguenti domande dalla 9 alla 19.
Se hai risposto **NO** alla domanda precedente, **salta** le domande dalla 9 alla 14 e **rispondi solo** alle domande dalla 15 alla 19.

ABITUDINI CORRELATE AGLI INTEGRATORI ALIMENTARI

9. Quali integratori alimentari usi attualmente oppure hai utilizzato in passato?
(è consentita più di una risposta)
□ Aminoacidi a catena ramificata (BCAA), Proteine in polvere, Creatina
□ Multivitaminici
□ Sport drinks
□ Prodotti dimagranti
□ Stimolanti (caffeina in capsule, guaranà, ginseng)
□ Altro (specificare) ...

10. Quante volte a settimana assumi oppure hai assunto integratori alimentari?
□ 1 □ 2 □ 3 □ 4 □ 5 □ 6 □7 □>7 (specificare ...)

11. Chi ha influenzato la tua decisione di utilizzare integratori alimentari?
(è consentita più di una risposta)

□ Nessuno
□ Allenatore
□ Amici/colleghi
□ Medico
□ Farmacista
□ Genitori
□ Internet
□ Pubblicità
□ Altro (specificare) ...

12. In che occasione assumi integratori alimentari?
□ In associazione all'attività sportiva
□ Periodicamente durante l'anno (ad esempio in associazione ai cambiamenti di stagione)
□ Quando avverto uno stato di astenia
□ Sporadicamente, senza una particolare cadenza

13. Come hai stabilito la quantità di integratore alimentare da assumere?
(è consentita più di una risposta)
□ Su consiglio del venditore
□ Su consiglio dell'allenatore
□ In base alle informazioni sulla scheda tecnica del prodotto
□ In base alle informazioni trovate in Internet
□ Calcolando la dose in base al peso corporeo
□ Arbitrariamente

14. Quali effetti collaterali (se presenti) hai percepito durante l'utilizzo di integratori alimentari?
(è consentita più di una risposta)
□ Nessuno
□ Nausea/vomito
□ Diarrea
□ Stipsi
□ Dolore addominale
□ Cefalea
□ Altro (specificare) ...

OPINIONI CORRELATE AGLI INTEGRATORI ALIMENTARI

15. Qual è la principale ragione per cui vengono utilizzati integratori alimentari?
(è consentita più di una risposta)
□ Per migliorare la performance sportiva
□ Per migliorare lo stato generale di salute e di benessere
□ Per aumentare il peso corporeo
□ Per ridurre il peso corporeo
□ Per sostituire un pasto
□ Per migliorare la performance mentale/cognitiva

16. Come giudichi l'effetto degli integratori alimentari quando assunti per il motivo che hai indicato sopra?
□ efficaci

□ piuttosto efficaci
□ piuttosto non efficaci
□ non efficaci

17. Su una scala da 1 a 10, come valuti l'impatto degli integratori alimentari sulla performance sportiva? (1 – nessun impatto, 10 - massimo impatto)

□1 □2 □ 3 □ 4 □5 □6 □ 7 □ 8 □9 □10

18. Su una scala da 1 a 10, come valuti la sicurezza degli integratori alimentari per la salute? (1 – completamente sicuri, 10 – completamente dannosi)

□1 □2 □ 3 □ 4 □5 □6 □ 7 □ 8 □9 □10

19. Indica il tuo livello di accordo o disaccordo con le seguenti affermazioni riguardanti gli integratori alimentari.

	**Assolutamente d'accordo**	**D'accordo**	**Neutrale**	**In disaccordo**	**Assolutamente in disaccordo**
**Gli integratori alimentari sono necessari in ogni fascia di età**					
**Gli integratori alimentari sono sicuri da usare**					
**L'assunzione regolare di integratori alimentari può prevenire malattie croniche**					
**L'assunzione regolare di integratori alimentari può prevenire patologie tumorali**					

Habits and beliefs related to food supplementation among Italian students

1. Age: ....years

2. Gender:

☐ Male

☐ Female

3. Education:

☐ University student - Medicine and Surgery programme

☐ University student - Health Sciences programme

☐ University student - another programme

☐ High school student

4. Have you practiced any sport for at least three consecutive months in the last 12 months?

□ Yes (please specify ...) □ No

5. If yes, how many practice sessions per week?

□ 1 □ 2 □ 3 □ 4 □ 5 □ 6 □ 7 □ >7 (specify …)

6. If yes, how long was an average practice session (in minutes)?

□ 30 □ 45 □ 60 □ 90 □ 120 □ >120 (specify ...)

7. Do you know what food supplements are?

□ Yes □ No

8. Have you ever consumed any type of food supplement?

□ Yes □ No

If you answered **YES** to the above question, please answer **all** the following questions **9-19**.
If you answered **NO** to the above question, please **skip** the questions **9-14** and **answer only** the questions **15-19**.

HABITS RELATED TO FOOD SUPPLEMENTATION

9. What types of food supplements have you consumed?
(more than one answer is allowed)

□ Branched-Chain Amino Acids (BCAAs), Proteins powder, Creatine

□ Multivitamins

□ Sport drinks

□ Weight-loss products

□ Stimulants (caffeine pills, guaranà, ginseng)

□ Other (please specify …)

10. How many times per week have you taken food supplements?

□ 1 □ 2 □ 3 □ 4 □ 5 □ 6 □7 □>7 (please specify………..)

11. Who influenced your decision to use food supplements?
(more than one answer is allowed)

□ No one
□ Trainer/Coach
□ Friend/coworker
□ Medical Doctor
□ Pharmacist
□ Parents
□ Internet
□ Media advertising
□ Other (please specify...)

12. Under what circumstances have you taken food supplements?
□ In association with sport activity
□ Periodically throughout the year (e.g. in association with season changes)
□ When I perceive physical weakness
□ Sporadically, without any particular pattern

13. How have you established the amount of food supplement you should take? (more than one answer is allowed)
□ Based on a seller's advice
□ Based on a trainer/coach's advice
□ Based on an indication in the product information leaflet
□ Found information on internet
□ Calculated based on body weight
□ Arbitrarily

14. What side effects (if any) have you experienced whilst taking food supplements? (more than one answer is allowed)
□ None
□ Nausea/vomiting
□ Diarrhoea
□ Constipation
□ Abdominal pain
□ Headaches
□ Other (please specify...)

BELIEFS RELATED TO FOOD SUPPLEMENTS

15. What is the purpose for consuming food supplements?
□ To enhance sport performance
□ To enhance overall health and wellness
□ To gain weight
□ To lose weight
□ To replace a skipped meal
□ To enhance mental/cognitive performance

16. In your opinion, how effective are food supplements, when taken for the purpose that you indicated above?
□ effective
□ rather effective
□ rather ineffective

□ ineffective

17. On a scale of 1 to 10, how would you rate the impact of food supplements on sport performance? (1 - no impact, 10 - the highest impact)
□1 □2 □ 3 □ 4 □5 □6 □ 7 □ 8 □9 □10

18. On a scale of 1 to 10, how would you rate the safety of food supplements? (1 - completely safe to health, 10 - extremely harmful to health)
□1 □2 □ 3 □ 4 □5 □6 □ 7 □ 8 □9 □10

19. Indicate to what level you agree or disagree with the following statements regarding food supplements:

	**Strongly agree**	**Agree**	**Neither agree nor disagree**	**Disagree**	**Strongly disagree**
**Food supplements are necessary at all ages**					
**Food supplements are essentially harmless**					
**Regular food supplement intake can prevent chronic diseases**					
**Regular food supplement intake can prevent cancer**					
